# Fever of Unknown Origin Unveiling Bone Marrow Necrosis Secondary to Diffuse Large B-cell Lymphoma

**DOI:** 10.7759/cureus.92492

**Published:** 2025-09-16

**Authors:** Ioannis Papakonstantinou, Despoina Liourdi, Leonidas Marinos, Nikolaos Papathanasiou, Athina-Lydia Mageiropoulou, Vasiliki Koletti, Panagiotis Zikos

**Affiliations:** 1 Department of Hematology, General Hospital of Patras, Patras, GRC; 2 Department of Hematopathology, Evangelismos General Hospital, Athens, GRC; 3 Department of Nuclear Medicine, General University Hospital of Patras, University of Patras, Patras, GRC; 4 Department of Internal Medicine, General Hospital of Patras, Patras, GRC

**Keywords:** bone marrow necrosis, dlbcl, fever of unknown origin, lymphoma, pet/ct

## Abstract

Bone marrow necrosis (BMN) is a very rare pathological finding that may arise in the context of numerous underlying conditions. It has been linked to hematologic malignancies, solid tumors, sickle cell disease, various infections, and certain medications. Advances in hematopathology have contributed significantly to the improved identification and diagnostic accuracy of BMN. We report a case of a 75-year-old woman who was evaluated for anemia and fever of unknown origin. Histopathological examination of a bone marrow biopsy revealed widespread necrosis accompanied by infiltration with diffuse large B-cell lymphoma (DLBCL). Positron emission tomography (PET)/computed tomography (CT) showed pathologic uptake in the skeletal system and in multiple lymph node regions, on both sides of the diaphragm. Following treatment with rituximab, cyclophosphamide, doxorubicin, vincristine, and prednisone (R-CHOP), a repeat bone marrow biopsy demonstrated persistent, complete BMN, whereas a subsequent PET/CT scan revealed complete metabolic response of the lymphoma, indicating a discordance between remission of lymphoma and the persistence of BMN. A comprehensive literature review was performed, and the clinical features of our patient were compared with data from previously published studies. The patient exhibited several characteristics commonly observed in the literature, including fever, anemia, and elevated lactate dehydrogenase (LDH) levels. However, the patient did not present with thrombocytopenia or a leukoerythroblastic peripheral smear. According to existing literature, the primary prognostic factor for patients with BMN is the effective control of the underlying disease. However, in our case, while remission of lymphoma was achieved, it was not associated with improvement in BMN. Areas for further discussion and advancement in the understanding of the etiology, diagnosis, and management of BMN are also highlighted.

## Introduction

A fever of unknown origin requires a comprehensive clinical assessment and extensive laboratory evaluation due to its broad differential diagnosis. Patients are typically admitted to internal medicine departments for initial management; however, depending on the evolving clinical and laboratory findings, multidisciplinary consultation with other specialties may be warranted. In some cases, uncommon or rare etiologies of fever are identified.

Bone marrow necrosis (BMN) is an uncommon pathologic finding that can be associated with fever [1,2], either as a direct clinical feature or as a consequence of the underlying disorder. A comprehensive bone marrow trephine biopsy assessment performed by hemopathologists, utilizing combined immunohistochemical panels, can detect BMN and may identify the underlying etiology. According to current literature, the primary causes of BMN include hematologic and nonhematologic malignancies, infections, and a broad spectrum of pharmacologic agents. 

Among hematologic malignancies, acute leukemias represent the most common cause of BMN [3], followed by non-Hodgkin lymphomas [1]. Early and accurate diagnosis enables the timely initiation of appropriate therapeutic interventions. Close collaboration among the internist, hematologist, and hemopathologist is essential for accurate diagnosis and effective management. Imaging studies can provide valuable information regarding the extent of BMN and may also assist in identifying optimal sites for biopsy. 

## Case presentation

A 75-year-old female patient, without notable personal or familial medical history, was admitted to the hospital with a one-month history of fever, reaching up to 38.5°C. She had recently completed a course of levofloxacin for a urinary tract infection. The patient reported no recent travel history and denied alcohol consumption and tobacco use. On physical examination, no peripheral lymph nodes were palpable. Laboratory investigations revealed significantly elevated C-reactive protein (CRP) and erythrocyte sedimentation rate (ESR), along with a marked increase in lactate dehydrogenase (LDH), and an elevated alkaline phosphatase. The patient was found to have microcytic anemia. White blood cell count and platelets were within the normal range (Table 1). Coagulation profile was within normal limits, including international normalized ratio (INR), activated partial thromboplastin time (aPTT), and fibrinogen levels. Serum ferritin was markedly elevated at 877 ng/mL (upper limit of normal: 150 ng/mL). Chest radiograph and abdominal ultrasound were unremarkable. Blood and urine cultures were obtained, and a transthoracic echocardiogram demonstrated no abnormalities. 

**Table 1:** Hematologic and biochemical workup MCV: mean corpuscular volume; LDH: lactate dehydrogenase; CRP: C-reactive protein; ESR: erythrocyte sedimentation rate

Laboratory exams	Initial values	End of treatment values	Normal range
Hemoglobin	9,0 g/dL	9,7 g/dL	12-16 g/dL
White blood cells	5,1 K/μL	4,8 K/μL	4-10 K/μL
Platelets	187 K/μL	161 K/μL	150-400 K/μL
MCV	72 fL	78 fL	80-97 fL
Alkaline phosphatase	251 IU/L	111 IU/L	<136 IU/L
LDH	1300 IU/L	217 IU/L	<230 IU/L
CRP	28 mg/dL	4 mg/dL	<0,5 mg/dL
ESR	67 mm/hr	24 mm/hr	<30 mm/hr

**Figure 1:**
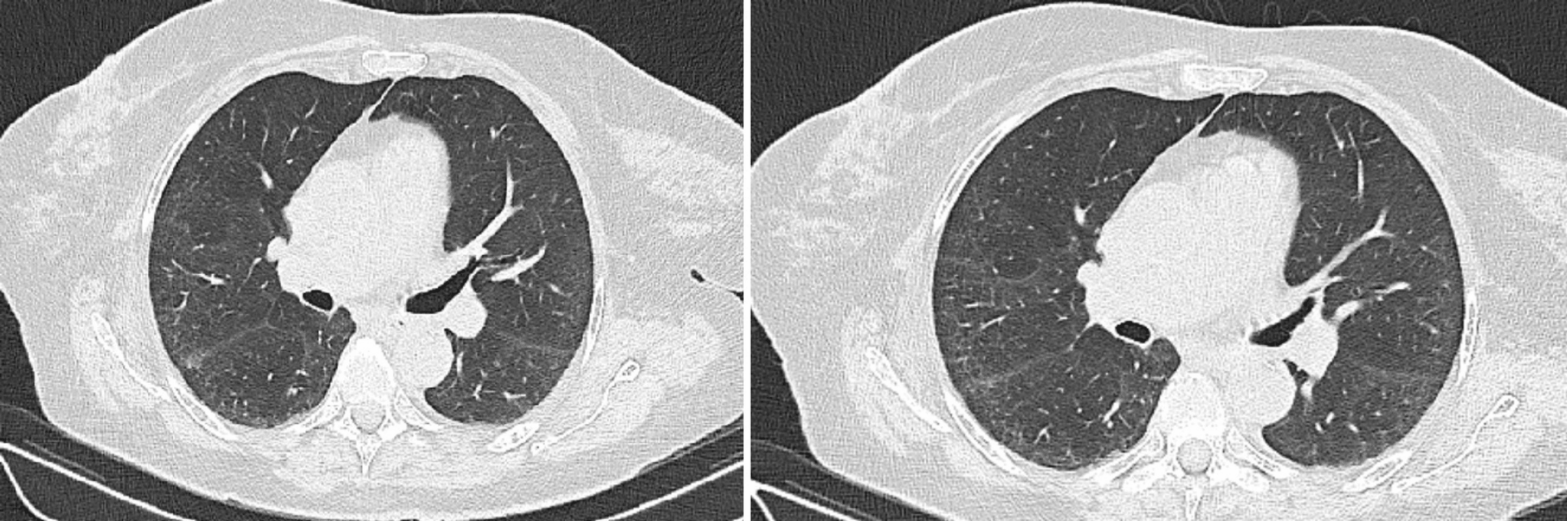
Computed tomography (CT) scan performed at the time of admission

A comprehensive infectious disease workup was initiated, including testing for *Leishmania*, *Leptospira*, *Brucella*, Epstein-Barr virus (EBV), cytomegalovirus (CMV), hepatitis B virus (HBV), hepatitis C virus (HCV), and human immunodeficiency virus (HIV), all of which were negative. A Mantoux test was also performed and yielded a negative result. Due to the persistence of fever, additional serologic testing was conducted for *Rickettsia*, *Coxiella*, *Borrelia*, *Bartonella*, *Mycoplasma*, and parvovirus. Computed tomography (CT) scan of the chest and abdomen revealed a mosaic attenuation pattern in the lungs, mediastinal lymph nodes measuring up to 13 mm, and axillary lymph nodes up to 8 mm (Figure 1). Mild hepatomegaly was noted, along with two hepatic cysts.

During hospitalization, the patient reported a sudden onset of numbness on the right side of her face. Neurological evaluation revealed no focal deficit. Given neurological symptomatology in the context of prolonged fever, a lumbar puncture was performed; cerebrospinal fluid analysis was within normal limits. The patient received broad-spectrum antimicrobial therapy, with piperacillin/tazobactam and vancomycin. In the subsequent days, vancomycin was substituted with linezolid, and doxycycline was added due to persistent febrile episodes, with the aim of broadening antimicrobial coverage against potential infectious agents. Despite adequate antibiotic coverage, the patient remained febrile during the first week of hospitalization. She also reported new-onset lumbar pain, which was not present at the time of admission.

Consultation with hematology and rheumatology was requested by the internal medicine team. A comprehensive clinical evaluation by the rheumatology consultant revealed no findings suggestive of an underlying rheumatologic disorder. Autoimmune serology panel, including antinuclear antibodies (ANA), anti-double-stranded DNA (anti-dsDNA), anti-neutrophil cytoplasmic antibodies (ANCA), anti-cyclic citrullinated peptide (anti-CCP), and extractable nuclear antigen (ENA) panel, was all negative.

The hematology team was consulted due to the presence of anemia, elevated lactate dehydrogenase (LDH), and persistent fever. Peripheral blood smear was unremarkable, except for the presence of microcytes. Flow cytometric immunophenotyping of peripheral blood did not identify blast cells or any clonal population indicative of a lymphoproliferative disorder or paroxysmal nocturnal hemoglobinuria. Quantitative immunoglobulin testing revealed hypogammaglobulinemia; however, serum protein electrophoresis and immunofixation did not demonstrate a monoclonal protein.

Given the clinical context, a bone marrow aspirate and biopsy were planned. However, multiple attempts at aspiration yielded a dry tap, precluding further analysis, including bone marrow immunophenotyping, cytogenetic studies, and microbial cultures. A bone marrow biopsy specimen was obtained and submitted to a specialized hematopathology laboratory for further evaluation. Concurrently, multiple sets of blood and urine cultures yielded negative results. 

**Figure 2:**
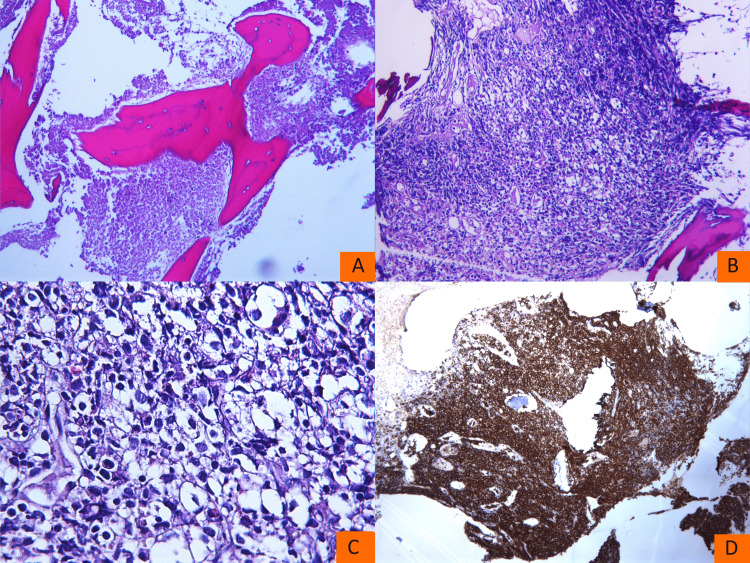
Bone marrow biopsy at diagnosis Area with bone marrow necrosis (A). Area with preserved bone marrow morphology (B). Higher magnification of the same area reveals large lymphoid cells (C). Positive CD20 staining (D)

Subsequent analysis by the hematopathologist initially revealed near-complete (80%) necrosis of the bone marrow in the biopsy specimen. Importantly, a few days later, it was also reported that limited preserved areas without necrosis were identified within the sample. These regions demonstrated infiltration by lymphoid cells consistent with a diagnosis of diffuse large B-cell lymphoma (DLBCL) (Figure 2). 

The infiltrated areas had complete (100%) replacement by large atypical lymphoid cells, many exhibiting centroblastic morphology. Immunohistochemical analysis revealed that the neoplastic cells were positive for CD20, Pax5, BCL6, and BCL2, with c-MYC expression in approximately 15% of the cells. The tumor cells were negative for CD30, CD15, MUM1, Cyclin D1, CD10, CD56, and CD34. Trilineage hematopoiesis was nearly absent, with marked depletion of erythroid, myeloid, and megakaryocytic precursors throughout the sample. Following consultation with the hematopathologist, and in light of the near-total necrosis observed in the specimen, with only focal areas of preserved tissue, a decision was made not to proceed with fluorescence in situ hybridization (FISH) analysis for c-MYC, BCL2, and BCL6 rearrangements. This decision was based on the concern that the results would likely be unreliable and not representative of the whole specimen, thereby limiting their diagnostic utility in evaluating for a possible double- or triple-hit lymphoma.

**Figure 3:**
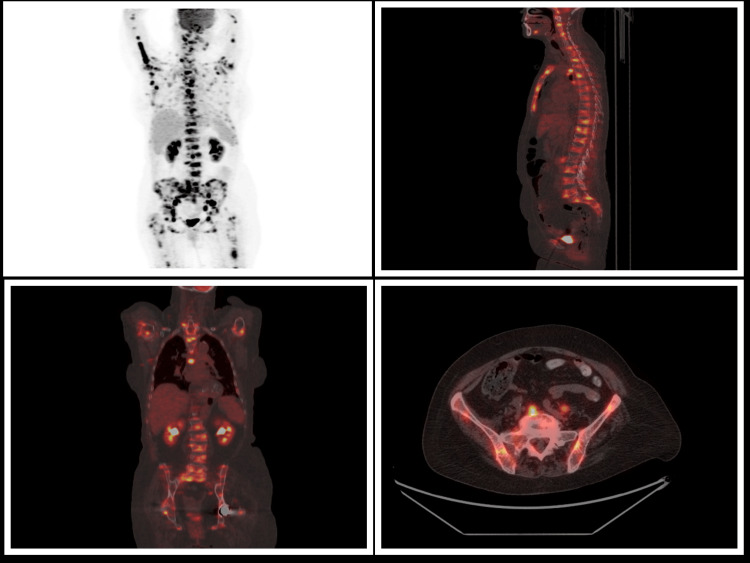
PET/CT shows pathologic uptake in the skeletal system and in multiple lymph node regions PET/CT: positron emission tomography/computed tomography

A PET/CT scan was performed for lymphoma staging. In addition to the anticipated intense fluorodeoxyglucose (FDG) uptake throughout the skeletal system, there was unexpectedly high metabolic activity in multiple lymph node regions. Notably, several cervical, supraclavicular, axillary, paratracheal, para-aortic, and hilar lymph nodes demonstrated markedly elevated FDG uptake, with SUVmax values reaching up to 22.7 (Figure 3).

Rituximab, cyclophosphamide, doxorubicin, vincristine, and prednisolone (R-CHOP) was the protocol selected for the treatment of DLBCL, with the patient becoming afebrile a week after the initiation of the protocol. Simultaneously, there was a rapid decrease in LDH values, and lumbar pain partially subsided. Initially, the first cycles were well-tolerated by the patient with early recovery of hematologic toxicity; however, her condition was later complicated by the onset of infections that required hospitalization, and mild cytopenias were observed. The differential diagnosis for these hematologic abnormalities included myelosuppression, potentially secondary to pharmacologic agents, primarily antibiotics, or attributable to the underlying infectious process. Additional considerations included persistent BMN or marrow infiltration in the context of refractory lymphoma, pending disease restaging.

At the end of the treatment plan, the patient showed persistent anemia with minimal improvement in hemoglobin levels. In contrast, complete normalization of LDH and alkaline phosphatase levels was noted, along with significant improvement in lumbar pain. The repeat bone marrow biopsy revealed complete BMN, precluding further morphological or immunohistochemical evaluation of the available material, therefore not allowing re-evalution of lymphoma. On the contrary, PET/CT, one month later, showed metabolic response. Differing from the initial PET/CT exam, lymph nodes showed no pathologic uptake. In addition, the skeletal system showed normal 18F-FDG uptake, with the exception of a small area in the body of the first lumbar vertebra with uptake similar to that of the mediastinal blood pool.

## Discussion

Dry tap as a first warning sign

In our patient, bone marrow aspiration from multiple sites along the posterior iliac crest resulted in a dry tap, a phenomenon observed in approximately 1.6% to 6.8% of bone marrow aspirates [4]. In the absence of technical errors, differential diagnosis for a dry aspiration includes idiopathic myelofibrosis, hairy cell leukemia, and diffuse bone marrow infiltration by malignant cells, which may originate from metastatic solid tumors or hematologic malignancies.

In cases of dry tap on bone marrow aspiration, histologic examination of the corresponding biopsy frequently reveals significant pathology, with normal marrow being identified in only a minority of cases. Common associated findings include the presence of peripheral nucleated red blood cells and thrombocytopenia [5]. In a study involving 2.768 bone marrow samples, 223 (8%) resulted in dry tap. Inadequate technique accounted for only 14 cases (6.3%). Hematologic malignancies were diagnosed in 73.5% of patients, benign hematologic disorders in 14.9%, and non-hematologic conditions in 4%. Thus, a dry tap on bone marrow aspiration is more commonly an indicator of underlying pathology than a technical failure [6].

History of BMN and associated conditions

Wade and Stevenson first described BMN in 1942 in a patient with sickle cell disease [1]. Since then, it has been linked to a wide range of underlying conditions and drugs. Although once considered an extremely rare diagnosis, increasing clinical awareness and the growing expertise of hematopathologists have led to more consistent recognition and accurate identification of this entity. Initially, BMN was primarily reported in autopsy cases; however, antemortem diagnoses are becoming increasingly documented. BMN results in the presence of “ghost cells” with atypical features in the nucleus and the cytoplasm, along with an amorphous eosinophilic-stained background. In 90% of cases, there is underlying neoplasia, hematological (60%) or solid (30%). The occurrence of BMN may arise at the time of diagnosis of the underlying disease, during treatment, or at the time of relapse.

The extent of BMN varies, ranging from limited to extensive [2].A grading scale has been proposed by Maisel et al., with three levels corresponding to the percentage of necrosis [7]. Grade I or mild necrosis affects less than 20% of bone marrow, Grade II or moderate affects 20-50%, and Grade III, affecting over 50% of bone marrow, is regarded as extensive. Our patient, with necrosis present in 80% of the available sample, is categorized into Grade III BMN. In cases of malignancy, necrosis is more often extensive when compared to benign causes.

Among hematologic malignancies, acute leukemias are the most prevalent cause [3,8,9], both acute lymphoblastic leukemia (ALL) and acute myeloid leukemia (AML). Lymphomas, mainly high-grade, represent the next most frequent cause, followed by rare cases of myeloproliferative neoplasms. BMN has also been described in association with HIV-related lymphoma [10]. Autologous stem cell transplantation has been successfully performed in non-Hodgkin lymphoma with extensive BMN [11]. In a review of five cases of BMN associated with DLBCL or high-grade B-cell lymphoma, necrosis was predominantly observed following chemotherapy [12]. In contrast, the current case describes the concurrent diagnosis of lymphoma and BMN, prior to the administration of any treatment. 

Various solid tumors have been associated with BMN, with gastric adenocarcinoma being frequently reported. Cases of necrosis have also been described in prostate, colon, and lung cancer [13], Ewing sarcoma, alveolar rhabdomyosarcoma, neuroblastoma, ductal breast carcinoma [12], and nasopharyngeal carcinoma [14]. Non-malignant causes include sickle cell disease, anorexia nervosa, disseminated intravascular coagulation (DIC), infections, hemolytic uremic syndrome (HUS), and antiphospholipid syndrome [15]. Additionally, case reports in the literature have documented BMN following treatment with agents such as all-trans retinoic acid [16,17], fludarabine, interferon-α, nivolumab [18], blinatumomab [19], imatinib [20], filgrastim [21], peg-filgrastim [22], and pegaspargase [23]. Occasionally, no probable cause of BMN is found, and these cases are characterized as idiopathic.

Some of these diseases and their association with BMN have been more extensively studied. ALL, one of the most common causes of BMN, may induce necrosis through the extrinsic pathway of apoptosis, as described in an analysis of seven patients [24]. In sickle cell disease, BMN may be complicated by fat embolism and multiorgan failure [25,26].

Τhe most prominent symptom in patients is bone pain, typically in areas of intense hematopoietic activity, primarily in the lumbar spine. Our patient did not present with bone pain upon admission; however, lumbar pain appeared in the following days. The next most common finding is fever, a feature our patient did exhibit. Regarding laboratory testing, anemia, thrombocytopenia, elevated LDH, and elevated alkaline phosphatase are important findings, while a leukoerythroblastic reaction may be present on the peripheral blood smear. Our patient had anemia, elevated LDH and alkaline phosphatase, but thrombocytopenia and a leukoerythroblastic smear were absent on admission.

Pathophysiology and differential diagnosis

Pathophysiology of BMN remains under investigation, as there is no common, definite mechanism for all cases. Hypoxemia and microcirculatory disruption in bone marrow appear as the most logical interpretation. Conditions like sickle cell crisis, antiphospholipid syndrome, and disseminated intravascular coagulation predispose to thrombosis and occlusion in the microvasculature. In cases of malignancy, the extensive infiltration of the bone marrow by neoplastic cells can interfere with its normal blood supply [27]. Drugs and high doses of radiation can cause direct toxicity to the bone marrow. The role of cytokines, with tumor necrosis factor (TNF) being the most prominent example, is being investigated in the pathogenesis of BMN. Endotoxins from specific pathogenic bacteria may also trigger the condition.

The first step toward the accurate diagnosis of BMN is to raise clinical suspicion. The provision of precise information from the clinician to the pathologist, as well as the latter’s experience, is of critical importance. Historical evidence has shown that when this condition is specifically investigated by a pathologist who includes it in their differential diagnosis, it is diagnosed much more frequently. The next important step is the investigation of the features that differentiate BMN from other conditions that present with certain similar histological characteristics [28]. The pathologist must distinguish BMN from avascular necrosis, idiopathic myelofibrosis, aplastic anemia, and amyloid deposition. BMN is characterized by loss of hematopoietic tissue and fat cells, with preserved cortical bone architecture. On the contrary, in avascular necrosis, damage to cortical bone exists. In aplastic anemia, hematopoietic marrow is replaced by adipocytes, whereas in amyloid deposition, Congo-red staining is positive.

BMN is a histopathological diagnosis that necessitates a bone marrow biopsy for confirmation. Nevertheless, magnetic resonance imaging (MRI) and PET/CT may offer valuable additional information [29,30]. BMN is characteristically identified by a diffuse, geographic distribution of marrow signal alteration, marked by a central area of variable intensity and a distinct, enhancing peripheral rim in MRI [31].

Prognosis

The prognosis of patients with BMN has historically been considered poor. However, more recent data indicate that this is not always the case, as prognosis largely depends on the underlying condition and the extent of necrosis. Moreover, the course of BMN and the response of the underlying disease to treatment do not always align. There are cases in which BMN may persist despite remission of the primary disease, and cases in which the necrosis may regress despite the presence of a resistant primary condition. In our case, BMN persisted on a repeat bone marrow biopsy, despite imaging findings indicating remission of the lymphoma. The continued presence of BMN may impair hematopoietic recovery, potentially resulting in sustained cytopenias, increased susceptibility to infections, and a possible need for transfusional support. This case underscores the importance of serial bone marrow evaluations to assess for persistent BMN, as imaging alone may be insufficient to fully characterize bone marrow status.

BMN is not always reversible, but in cases where necrosis resolves, repopulation with hematopoietic cells is possible, with bone marrow occasionally exhibiting fibrous scars. Although this reparative process can proceed rapidly in some cases [32], it typically requires a more prolonged period for resolution. The aforementioned peripheral rim observed on MRI may, in certain instances, correspond to regions with relatively preserved bone marrow or areas undergoing early stages of hematopoietic regeneration.

Enhancing the diagnostic accuracy of BMN

An extensive immunohistochemical panel is needed in cases where complete BMN prevents identification of the underlying disease. A proposed panel currently includes Grocott’s methenamine silver (GMS) stain; acid-fast bacillus (AFB) stain; immunomarkers CD3, CD20, CD34, and CD117; and pan-cytokeratin [33]. This panel is designed to detect a broad spectrum of common infectious and neoplastic conditions associated with BMN. In cases where a definitive diagnosis remains elusive, a comprehensive review of drug exposure, an extended infectious disease panel, and additional immunohistochemical studies of available specimens may aid in establishing the diagnosis.

## Conclusions

Numerous questions concerning patients with BMN remain unresolved. Specifically, the exact pathophysiological mechanism of BMN requires further clarification. Furthermore, the potential for identifying specific biomarkers for the diagnosis and monitoring of necrosis remains an area of interest. Broader use of imaging techniques is also crucial for obtaining targeted biopsy samples. Regarding treatment, a promising area of investigation involves the exploration of pharmacological agents that may promote the recovery of affected bone marrow, potentially through mechanisms involving anti-inflammatory or antithrombotic actions.

Another unresolved question is whether BMN should impact the therapeutic strategy for the underlying disease, and if so, how. One perspective is that it should be considered as an additional aggressive manifestation of the disease, thereby necessitating a more intensive treatment approach. Conversely, the coexistence of BMN with infiltration by the primary disease may increase the patient’s susceptibility to higher-grade cytopenias during treatment, suggesting that a less aggressive treatment protocol may be more appropriate.
